# Investigation of Antimicrobial Resistance in *Escherichia coli* and Enterococci Isolated from Tibetan Pigs

**DOI:** 10.1371/journal.pone.0095623

**Published:** 2014-04-18

**Authors:** Peng Li, Dongfang Wu, Kunyao Liu, Sizhu Suolang, Tao He, Xuan Liu, Congming Wu, Yang Wang, Degui Lin

**Affiliations:** 1 Department of Small Animal Clinical Sciences, College of Veterinary Medicine, China Agricultural University, Beijing, P. R. China; 2 National Center for Veterinary Drug Safety Evaluation, College of Veterinary Medicine, China Agricultural University, Beijing, P. R. China; 3 Department of Pharmaceuticals, China Institute of Veterinary Drugs Control, Beijing, P. R. China; 4 College of Agricultural and Animal Husbandry, Tibet University, Linzhi, P. R. China; U. S. Salinity Lab, United States of America

## Abstract

**Objectives:**

This study investigated the antimicrobial resistance of *Escherichia coli* and enterococci isolated from free-ranging Tibetan pigs in Tibet, China, and analyzed the influence of free-ranging husbandry on antimicrobial resistance.

**Methods:**

A total of 232 fecal samples were collected from Tibetan pigs, and the disk diffusion method was used to examine their antimicrobial resistance. Broth microdilution and agar dilution methods were used to determine minimum inhibitory concentrations for antimicrobial agents for which disks were not commercially available.

**Results:**

A total of 129 *E. coli* isolates and 84 *Enterococcus* isolates were recovered from the fecal samples. All *E. coli* isolates were susceptible to amoxicillin/clavulanic acid, and 40.4% were resistant to tetracycline. A small number of isolates were resistant to florfenicol (27.9%), ampicillin (27.9%), sulfamethoxazole/trimethoprim (19.4%), nalidixic acid (19.4%), streptomycin (16.2%) and ceftiofur (10.9%), and very low resistance rates to ciprofloxacin (7.8%), gentamicin (6.9%), and spectinomycin (2.3%) were observed in *E. coli*. All *Enterococcus* isolates, including *E. faecium*, *E. faecalis*, *E. hirae*, and *E. mundtii*, were susceptible to amoxicillin/clavulanic acid and vancomycin, but showed high frequencies of resistance to oxacillin (92.8%), clindamycin (82.1%), tetracycline (64.3%), and erythromycin (48.8%). Resistance rates to florfenicol (17.9%), penicillin (6.0%), ciprofloxacin (3.6%), levofloxacin (1.2%), and ampicillin (1.2%) were low. Only one high-level streptomycin resistant *E. faecium* isolate and one high-level gentamicin resistant *E. faecium* isolate were observed. Approximately 20% and 70% of *E. coli* and *Enterococcus* isolates, respectively, were defined as multidrug-resistant.

**Conclusions:**

In this study, *E. coli* and *Enterococcus* isolated from free-ranging Tibetan pigs showed relatively lower resistance rates than those in other areas of China, where more intensive farming practices are used. These results also revealed that free-range husbandry and absence of antibiotic use could decrease the occurrence of antimicrobial resistance to some extent.

## Introduction

*Escherichia coli* is a commensal bacterium and opportunistic pathogen that is commonly found in the intestinal tracts of animals and humans [1]. Because it is regarded as an indicator organism of antimicrobial resistance for a wide range of bacteria [2], data on antimicrobial resistance of *E. coli* is available from many countries [3]–[6], including the northern, central, and southern areas of China [7]. Enterococci are Gram-positive bacteria that are also commonly found in the gastrointestinal tracts of animals and humans. *Enterococcus* species, especially *E. faecium* and *E. faecalis*, are responsible for many different infections in humans and animals, including urinary tract and wound infections, bacteremia, and endocarditis [8]. The increasing resistance of *Enterococcus* species to multiple antimicrobial agents, particularly the appearance of vancomycin-resistant *Enterococcus*, makes treatment of enterococcal infections progressively more difficult. Therefore, many studies have focused on antimicrobial resistance of *Enterococcus* species [9]–[11], including in China [12]. Because of the importance of antimicrobial resistance in bacteria of food animal origin, the majority of these studies used isolates collected from intensive farms. However, few data are available for free-ranging livestock, especially in China. To examine the antimicrobial resistance of *E. coli* and enterococcal isolates collected from free-ranging livestock in their natural environment, the Tibetan pig was chosen for investigation in this study.

The Tibetan pig, which has black skin, coarse hair, small erect ears, a short trunk, and plentiful muscle, is widely known for its tolerance to disease and strong adaptability to the harsh Tibetan environment of low oxygen levels and changeable temperatures [13]. More importantly, the Tibetan pig is free-ranging in its natural environment with adequate water and food, and no antimicrobials are used for therapy or growth promotion. To our knowledge, there are no reports on antimicrobial resistance of bacteria of Tibetan pig origin. In this study, we aimed to evaluate the susceptibility of *E. coli* and enterococci isolated from Tibetan pigs to 11 and 13 antibiotics, respectively, which are commonly used in human and veterinary medicine. We also compared the resistance rates of these isolates with rates from other areas of China that use modern intensive farming practices, in which antimicrobials are frequently administered.

## Materials and Methods

### Ethics Statement

This study was approved by the Beijing Municipality Review of Welfare and Ethics of Laboratory Animals (BAOLA 2005). No associated permit number was required, for both commercial animals and free-ranging animals sampling were approved. No specific permission was required for the sampling location, and we confirm that the field study did not involve endangered or protected species. In addition, animal sampling practices were carried out in strict accordance with the principles of the BAOLA (2005). Furthermore, samples were collected from freshly voided feces, rather than rectal swabbing of the pigs, and all efforts were made to minimize any animal suffering.

### Sample collection, isolation, and identification

A total of 232 fecal samples were collected from Tibetan pigs (6–12 months of age) from June to October, 2012. The animals were grazed on a semi-mountainous region approximately 5 km from Jue Mugou village (latitude 29.66, longitude 94.30), Bayi Town in Linzhi County, Tibet. This area has an average altitude of 3,100 m. Importantly, antimicrobials were not used as either therapeutic agents or as growth promoters in these animals. Fresh fecal samples were collected from near the animals using sterile cotton swabs, which were immediately put into sterile containers and transported on ice to the laboratory. All samples were simultaneously streaked on chromogenic medium (CHROMagar, Zhengzhou, China) for isolation of *E. coli* (ECC medium) and *Enterococcus* medium base (Land Bridge, Beijing, China) supplemented with ammonium ferric citrate for isolation of enterococci, and incubated at 37°C for 24 h. At least one suspected colony per plate was selected and stored in 20% glycerol at −80°C.

For species identification, 16S rDNA sequencing was performed using universal primers as previously described [14], and the PCR amplicons were sequenced by GENEWIZ (Beijing, China). Obtained sequences were subjected to BLAST analysis in the GenBank database (http://blast.ncbi.nlm.nih.gov/Blast.cgi), and species were identified on the basis of nucleotide similarities ≥99.6%. The identification results were further confirmed using the API-20E system or Rapid ID 32 Strep system (bioMerieux, Craponne, France).

### Antimicrobial susceptibility testing

Antimicrobial susceptibility of *E. coli* isolates was determined using the disk diffusion and broth microdilution methods, according to the standards and interpretive criteria described by the Clinical and Laboratory Standards Institute (CLSI) [15]. The disk diffusion method was performed on Mueller-Hinton agar (Land Bridge), using the following antimicrobials (all from Oxoid, Beijing, China): aminoglycosides (spectinomycin, gentamicin, streptomycin), β-lactams (ampicillin, amoxicillin/clavulanic acid), fluoroquinolones (ciprofloxacin, nalidixic acid), sulfamethoxazole/trimethoprim, and tetracycline. The minimum inhibitory concentrations (MICs) of florfenicol and ceftiofur (China Institute of Veterinary Drug Control, Beijing, China), for which commercial disks are not available, were determined using the broth microdilution method [15]. *E. coli* ATCC 25922 was used as the quality control strain.

*Enterococcus* isolates were tested by the disk diffusion method for their susceptibility to the following 11 antimicrobials: β-lactams (amoxicillin/clavulanic acid, oxacillin, ampicillin, penicillin), fluoroquinolones (levofloxacin, ciprofloxacin), clindamycin, tetracycline, and erythromycin. Susceptibility to florfenicol and vancomycin was examined using the broth microdilution method, using the protocol described above. In addition, an agar screening method was used to detect high-level streptomycin resistance (HLSR) and high-level gentamicin resistance (HLGR), according to the method of the CLSI [15]. *E. faecalis* ATCC 29212 was used as the quality control strain.

Multidrug-resistant (MDR) *E. coli* or enterococcal isolates were defined as those that were resistant to at least three different classes of the antimicrobial agents tested. Tested concentrations and breakpoints of each antimicrobial for *E. coli* and *Enterococcus* species are shown in Table 1 and Table 2, respectively.

**Table 1:** Antimicrobial susceptibility testing and antimicrobial resistance parameters of *E. coli*.

Antimicrobial agents	Method	Disk content (µg) or test range (µg ml^−1^)	Resistance breakpoint (millimeters or µg ml^−1^)	No. of resistant and MDR isolates (%)
AMP		10	≤13	36(27.9)
A/C		20/10	≤13	0(0)
SH	disk	100	≤10	3(2.3)
CN	diffusion	10	≤12	9(6.9)
STR		10	≤11	21(16.2)
CIP		5	≤15	10(7.8)
NAL		30	≤13	25(19.4)
SXT		1.25/23.75	≤10	25(19.4)
TET		30	≤11	52(40.4)
FFC*	broth	0.125–64	≥32	36(27.9)
CEF*	microdilution	0.125–64	≥2	14(10.9)
			MDR	32(24.8)

AMP: ampicillin; A/C: amoxicillin/clavulanic acid; SH: spectinomycin; CN: gentamicin; STR: streptomycin; CIP: ciprofloxacin; NAL: nalidixic acid; SXT: sulfamethoxazole/trimethoprim; TET: tetracycline; FFC: florfenicol; CEF: ceftiofur.

*No breakpoints were available for *E. coli* in the CLSI documents. In this study, ≥32 µg ml^−1^ was the tentative breakpoint for florfenicol, and ≥2 µg ml^−1^ was used for ceftiofur, according to EUCAST (http://www.eucast.org/mic_distributions/).

**Table 2:** Antimicrobial susceptibility testing and antimicrobial resistance parameters of *Enterococcus* species.

Antimicrobial agents	Method	Disk content (µg) or test range (µg ml^−1^)	Resistance breakpoint (millimeters or µg ml^−1^)	Prevalence of resistant/MDR isolates (%) per species				
				*E. faecium* (n = 45)	*E. faecalis* (n = 31)	*E. hirae* (n = 5)	*E. mundtii* (n = 3)	Total
A/C		20/10	≤13	0(0)	0(0)	0(0)	0(0)	0(0)
OXA		1	≤10	40(88.9)	31(100)	4(80.0)	3(100)	78(92.8)
AMP	disk	10	≤16	1(2.2)	0(0)	0(0)	0(0)	1(1.2)
PEN	diffusion	10	≤14	4(8.9)	0(0)	1(20.0)	0(0)	5(6.0)
CIP		5	≤15	3(6.7)	0(0)	0(0)	0(0)	3(3.6)
LEV		5	≤13	1(2.2)	0(0)	0(0)	0(0)	1(1.2)
ERY		15	≤13	9(20.0)	29(93.5)	2(40.0)	1(33.3)	41(48.8)
CLI		2	≤14	32(71.1)	30(96.8)	4(80.0)	3(100)	69(82.1)
TET		30	≤14	20(44.4)	29(93.5)	2(40.0)	1(33.3)	54(64.3)
FFC*	broth	0.125–64	≥8	7(15.6)	5(16.1)	2(40.0)	1(33.3)	15(17.9)
VAN	microdilution	0.5–256	≥32	0(0)	0(0)	0(0)	0(0)	0(0)
HLSR	agar	2000	>1 colony	1(2.2)	0(0)	0(0)	0(0)	1(1.2)
HLGR	screening	500	>1 colony	1(2.2)	0(0)	0(0)	0(0)	1(1.2)
			MDR	22(48.9)	30(96.8)	3(60.0)	1(33.3)	56(66.7)

OXA: oxacillin; PEN: penicillin; LEV: levofloxacin; ERY: erythromycin; CLI: clindamycin; VAN: vancomycin; HLSR: high-level resistance to streptomycin; HLGR: high-level resistance to gentamicin.

*No breakpoints were available for *Enterococcus* in the CLSI documents. In this study, ≥8 µg ml^−1^ was the tentative breakpoint.

### Statistical analysis

The antimicrobial resistance rates of *E. coli* and *Enterococcus* species were compared, respectively, with other similar studies using the chi-squared test. A *P*-value of less than 0.05 was considered significant. Differences in the frequency of antimicrobial resistance between different species of *Enterococcus* were analyzed in the same way.

## Results

### Bacterial identification

A total of 129 *E. coli* isolates and 84 *Enterococcus* isolates were identified from 232 collected fecal samples. The isolation rates for *E. coli* and *Enterococcus* were 55.6% and 36.2%, respectively. Among the 84 *Enterococcus* isolates, four different species were identified: *E. faecium* (45/84, 53.6%), *E. faecalis* (31/84, 36.9%), *E. hirae* (5/84, 6.0%), and *E. mundtii* (3/84, 3.6%).

### Antimicrobial resistance of the isolates

All 129 *E. coli* isolates were susceptible to amoxicillin/clavulanic acid (Table 1). For other β-lactam antibiotics, including ceftiofur and ampicillin, the frequency of resistance was less than 30% (10.9% and 27.9%, respectively). Less than 10% of *E. coli* isolates were resistant to ciprofloxacin (7.8%), gentamicin (6.9%), or spectinomycin (2.3%). Approximately 20% of the isolates showed resistance to sulfamethoxazole/trimethoprim (19.4%), nalidixic acid (19.4%), or streptomycin (16.2%), and resistance to florfenicol was 27.9%. Approximately 40% of the isolates were resistant to tetracycline (40.4%), which was the highest rate of resistance among all of the tested antimicrobial agents. Furthermore, 24.8% of the *E. coli* isolates were resistant to more than three antimicrobial agents.

None of 84 *Enterococcus* isolates were resistant to amoxicillin/clavulanic acid or vancomycin (Table 2), but more than 60% were resistant to oxacillin, clindamycin, or tetracycline (92.8%, 82.1%, and 64.3%, respectively). The rate of resistance to erythromycin was nearly 50%, and 17.9% of the isolates were resistant to florfenicol. However, *Enterococcus* isolates showed low rates of resistance to some of the other tested antimicrobials, as follows: penicillin (6.0%), ciprofloxacin (3.6%), levofloxacin (1.2%), and ampicillin (1.2%). In addition, in tests for high-level resistance to streptomycin and gentamicin, only one *Enterococcus* isolate tested positive in each of the respective assays. Approximately 66.7% of the enterococci were defined as multidrug-resistant isolates.

Among the *Enterococcus* isolates, only *E. faecium* isolates showed resistance to ciprofloxacin, levofloxacin, or ampicillin, as well as presenting high-level resistance to streptomycin and gentamicin. Overall, the majority of the *Enterococcus* isolates were resistant to oxacillin and clindamycin, with the highest rates of resistance observed for *E. faecalis* (100% to oxacillin) and *E. mundtii* (100% to clindamycin and oxacillin). Although there were relatively high rates of resistance to tetracycline and erythromycin, differences between *E. faecalis* and *E. faecium* were significant (*P*<0.05). Interestingly, the rates of resistance to florfenicol in *E. faecalis* and *E. faecium* (16.1% and 15.6%, respectively) were lower than those of *E. hirae* and *E. mundtii* (40% and 33.3%, respectively). All *E. faecalis* and *E. mundtii* isolates were susceptible to penicillin, while *E. faecium* and *E. hirae* isolates showed some resistance (8.9% and 20.0%, respectively). The multidrug resistance rates from highest to lowest were as follows: *E. faecalis* (96.8%), *E. hirae* (60.0%), *E. faecium* (48.9%), and *E. mundtii* (33.3%), and the significant difference (P<0.05) was only observed between *E. faecalis* and *E. faecium*.

## Discussion

In this study, we examined the rates of antimicrobial resistance in *E. coli* and *Enterococcus* species isolated from the feces of free-ranging Tibetan Pigs that had not been exposed to antibiotics. We determined that the rates of antimicrobial resistance of *E. coli* isolates to most of the tested agents were lower than those found in a previous study conducted in Southern, Central, and Northern China [7], where intensive farming is more common. Higher rates of resistance to nalidixic acid, spectinomycin, and sulfamethoxazole/trimethoprim have also been reported from intensive farms in other countries, compared with the rates found in the current study [16]–[17]. Tetracycline was widely used in the past to treat bacterial diseases in pigs [18], and a previous study in Shandong, China, detected high concentrations of tetracycline in swine manure as a result of extensive prophylactic use of tetracycline [19]. In addition, natural water was considered a possible mean of antimicrobial resistance dissemination [20], and previous studies have reported the presence of tetracycline-resistant genes in river basins in China [21]–[23]. Besides, tetracycline resistance was also determined in soils in different cities in China [24]. Furthermore, high abundance of tetracycline resistance genes were confirmed in human gut microbiota in China [25]. The sampling location in this study is a tourism hotspot with great human mobility, which may result in the tetracycline resistance gene or bacteria released from human beings into the environment (water and soil) in Tibet. Taken together, all of these studies perhaps partly explained the relatively high frequency of tetracycline resistance in *E. coli* of Tibet pigs origin. Resistance rates of *E. coli* in the present study to ceftiofur, streptomycin, and gentamicin were also lower than rates in *E. coli* isolated from chickens whose feed was supplemented with the corresponding antimicrobials [26]. The reason for these differences in antimicrobial resistance rates may be the increased use of antibiotics as therapeutic agents and growth promoters in intensive farming practices. Interestingly, another study in Spain [27] demonstrated that *E. coli* isolated from wild, free-ranging boars, in where the use of antimicrobials can be ruled out, exhibited much lower resistance to a wide variety of antimicrobial agents, including ciprofloxacin (3.2%), ampicillin (4.8%), tetracycline (7.9%), streptomycin (4.8%), and nalidixic acid (1.6%). This variation in resistance rates between free-ranging and intensively-farmed animals may also be linked to the proximity of the animals to human populations, environmental conditions, and livestock density, although the influence of these factors requires further investigation.

In this study, the predominance of *E. faecium* and *E. faecalis* among the *Enterococcus* isolates is consistent with previous studies on pigs in China [12]. However, other species that were found in previous studies, such as *E. casseliflavus*, *E. durans*, and *E. gallinarum* [11]–[12], were not isolated in the current study. *E. avium*, which was also not present in the feces of the Tibetan pigs, has previously been isolated from domestic pets [28]. Interestingly, *E. mundtii*, which was present in the current samples, was not isolated in those previous studies. The reason for this diversity may be attributed to differences in geographical location, animal species, and the living environment.

Vancomycin is often regarded as the last line of defense in the treatment of many bacterial pathogens [29]. In recent years, vancomycin-resistant *Enterococcus* strains, which also show resistance to several other antibiotics used in clinical practice, have emerged [30]. These strains represent a global threat to public health. No vancomycin-resistant *Enterococcus* isolates were found in this study. Enterococci are intrinsically resistant to clindamycin and erythromycin [29], [31], which was supported by the results of the current study. All *Enterococcus* species showed resistance to clindamycin, while *E. faecalis* isolates showed higher resistance rates to erythromycin than the other three enterococcal species, which was consistent with previous reports from Beijing and Shandong Province [12]. In addition, enterococci are often naturally resistant to β-lactam antibiotics [32]. However, a previous study on domestic pets showed low resistance rates for amoxicillin/clavulanic acid in *Enterococcus* isolates [28], and the *Enterococcus* isolates collected in the current study were also susceptible to this antibiotic. The observed resistance rates to ampicillin and penicillin were also lower than those in a previous study conducted in farms in Beijing, China [12], where the animals were free-ranging, but a wide variety of antibiotics, such as penicillins, aminoglycosides and phenicols, were used for disease treatment and prevention. Thus, frequent use of antibiotics in Beijing farms may be the primary reason for higher rates of penicillin-resistant and ampicillin-resistant isolates. *Enterococcus* strains are considered to be naturally resistant to oxacillin [30]. In the current study, oxacillin was the only β-lactam antibiotic to which all *Enterococcus* species showed high levels of resistance, which supports an earlier study [28] that showed 100% of *Enterococcus* isolates were resistant to oxacillin. Compared with bacteria isolated from domestic pets [28], rates of resistance to ciprofloxacin and tetracycline for *Enterococcus* isolates in this study were low, perhaps as a result of the frequent use of antimicrobials in pets. In addition, one *E. faecium* isolate was resistant to high-level streptomycin and one *E. faecium* isolate showed resistance to high-level gentamicin in this study, while previous studies from Beijing and Shandong Province, China [12], South Korea [33], and Canada [34] showed higher resistance rates to these aminoglycosides in both *E. faecium* and *E. faecalis* isolates. The major reason for the high resistance rates to high-level streptomycin and gentamicin treatment is that aminoglycoside antibiotics are commonly used as therapeutic and growth promotion agents in veterinary medicine in those areas.

## Conclusions

Overall, owing to absence of antibiotic use, *E. coli* and enterococci isolated from free-ranging pigs showed lower resistance levels to the majority of antibiotics compared with bacteria isolated from intensively-farmed animals in China and other countries. Our findings revealed that free-ranging husbandry, along with restricting prophylactic use of antibiotics, may decrease the occurrence of antimicrobial resistance to some extent.
